# A novel log odds of positive lymph nodes–based nomogram for predicting overall survival in patients with colorectal signet ring cell carcinoma: a SEER population-based study

**DOI:** 10.1007/s00384-024-04622-x

**Published:** 2024-04-01

**Authors:** Wenqian Yu, Boqi Xu, Peng Li

**Affiliations:** 1 Department of Gastrointestinal Surgery, Affiliated Hospital of Nantong University, Chongchuan District, No. 20 Xisi Road, Nantong, 226000 China; 2 Medical School, Nantong University, Nantong, Jiangsu Province, China; 3 Department of General Surgery, The First Affiliated Hospital of Soochow University, Suzhou, China

**Keywords:** Colorectal cancer, Signet ring cell carcinoma, Nomogram, LODDS, Lymph node stage, SEER database

## Abstract

**Purpose:**

Considering the poor prognosis and high lymph node (LN) involvement rate of colorectal signet ring cell carcinoma (SRCC), this study aimed to construct a prognostic nomogram to predict overall survival (OS) with satisfactory accuracy and utility, based on LN status indicators with superior predictability.

**Methods:**

Using the Surveillance, Epidemiology, and End Results (SEER) database, we obtained cases of colorectal SRCC patients and employed univariate and multivariate Cox analyses to determine independent prognostic factors. Kaplan–Meier curves were utilized to visualize survival differences among these factors. Receiver operating characteristic curves were generated to assess predictive performances of models incorporating various LN status indicators. A novel nomogram, containing optimal LN status indicators and other prognostic factors, was developed to predict OS, whose discriminatory ability and accuracy were evaluated using calibration curves and decision curve analysis.

**Results:**

A total of 1663 SRCC patients were screened from SEER database. Older patients and those with grades III–IV, tumor sizes > 39 mm, T3/T4 stage, N1/N2 stage, M1 stage, and higher log odds of positive lymph nodes (LODDS) values exhibited poorer prognoses. Age, grade, tumor size, TNM stage, and LODDS were independent prognostic factors. The model containing N stage and LODDS outperformed the one relying solely on N stage as LN status indicator, resulting in a validated nomogram for accurately predicting OS in SRCC patients.

**Conclusion:**

The integration of LODDS, N stage, and other risk factors into a nomogram offered precise OS predictions, enhancing therapeutic decision-making and tailored follow-up management for colorectal SRCC patients.

## Introduction

Colorectal cancer (CRC) ranks as the third most common malignancy and the third leading cause of cancer-related death in the United States. Significantly, the number of men younger than 50 who are afflicted with CRC is increasing at an alarming rate [1]. Initially proposed by Saphir and Laufman in 1951 [2], signet ring cell carcinoma (SRCC) is a distinct subtype of CRC, one that is inherently composed of no less than 50% of tumorous cells displaying the morphological features of signet ring cells, accounting for approximately 1% of all CRC cases [3]. Apart from aggressive behaviors including larger tumor size, more advanced tumor stages at initial diagnosis, and higher incidence of peritoneal dissemination, SRCC also has a higher percentage of lymph node metastasis (LNM) [3], making the accurate diagnosis and stratification crucial to the selection of treatment especially regarding chemotherapy. However, the American Joint Committee on Cancer (AJCC) tumor-node-metastasis (TNM) staging system, a widely adopted assessment for LNM, aroused controversy, as its reliability can be adversely affected by the number of lymph nodes (LNs) dissected, the extent of lymph node (LN) dissection, individual differences in the pattern of regional LNM, and the surgeon’s skill [4].

In recent years, several studies have constructed a predictive model for survival rates of colorectal SRCC patients [5] but simply using N stage as the LN prognostic factor, which lacks accuracy. Liang et al. proposed several LN prognostic factors, including positive lymph nodes (PLN), the lymph node ratio (LNR), and log odds of positive lymph nodes (LODDS), to estimate the prognosis of colorectal patients [6]. However, there is no published research utilizing novel LNM indicators for predicting the prognosis of SRCC patients.

Due to the worse prognosis and the differences in response to the common therapeutic schedules, a better classification of this rare histological subtype is needed [7]. The present study aimed to construct a prognostic nomogram with satisfactory accuracy and utility, based on LN status indicators with superior predictability. Therefore, we compared the predictive values among different LN status indicators of SRCC patients by analyzing data from the Surveillance, Epidemiology, and End Results (SEER) database, established a novel nomogram incorporating LN status indicators which showed the best predictive performance for survival rates, and validated the nomogram in internal validation cohorts.

## Materials and methods

### Data source

This study adhered to the TRIPOD statement. The data utilized in this study were collected from the program SEER*Stat (Version 8.4.0.1) grounded on Incidence-SEER Research Plus Data, 17 Registries, Nov 2021 Sub(2000–2019), delivering comprehensive clinicopathological data. The SEER database covers approximately 48% of the United States population, with information from 18 states that represent all regions of the country [8]. Given the anonymous nature of the data available in the SEER database, the requirement for informed consent was waived in this study.

### Study population

**Fig. 1:**
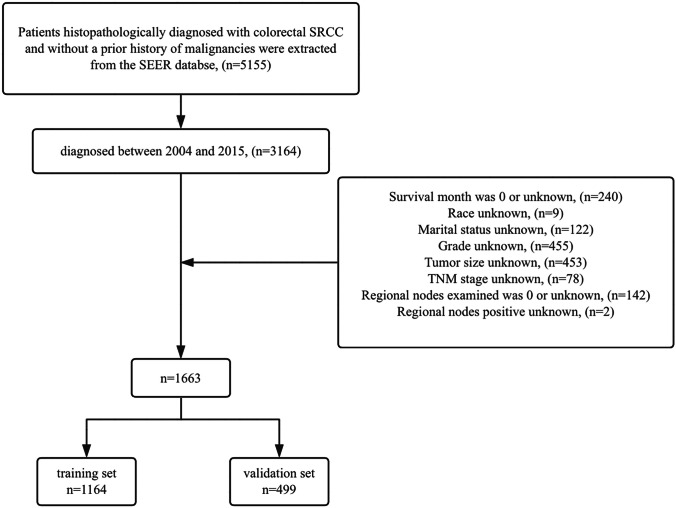
Flowchart illustrating patient selection of this study. Abbreviations: SEER, the Surveillance, Epidemiology, and End Results

Patients with primary tumor site labeled as C18.2, C18.3, C18.4, C18.5, C18.6, C18.7, C18.9, C19.9, and C20.9 were included in the study. Eligible patients were those with histologically confirmed SRCC (code: 8490/3) and without a prior history of malignancies between 2004 and 2015. Exclusion criteria encompassed the following: (1) patients with unknown survival duration or those who died within 1 month post-surgery (indicated by a survival duration of 0 months); (2) patients lacking sufficient clinical information (unknown age, race, marital status, grade, tumor size, TNM stage, chemotherapy status, radiotherapy status, regional LNs examined, regional LNs positive). Tumor grades were dichotomized into grades I–II and grades III–IV. T and N stages were categorized according to the 6th edition of the AJCC TNM staging system between 2004 and 2015. The process is shown schematically in Fig. 1.

### Identifications of cutoff values for variables

The LNR was calculated as the proportion of PLN to the total number of examined LNs (ELN). LODDS, on the other hand, was derived using the formula log [(PLN + 0.05) / (ELN − PLN + 0.05)]. Utilizing X-tile software (version 3.6.1; Yale University, New Haven, CT, USA), the data pertaining to tumor size, PLN, LNR, and LODDS were stratified into two distinct groups based on overall survival (OS).

### Statistical analysis

All statistical analyses were executed in R software version 4.4.2 (Institute for Statistics and Mathematics, Vienna, Austria; https://www.r-project.org/). The primary endpoint was the OS rate, defined as the duration between the diagnosis of SRCC and death from any cause. The study cohort was randomly divided into a training set and a validation set at a ratio of 7:3. Within the training set, univariate Cox regression analysis was initially conducted to pinpoint pertinent prognostic factors (*p* < 0.05), succeeded by multivariate Cox regression analysis to ascertain the independent prognostic factors. Kaplan–Meier curves demonstrated marked differences in OS rates concerning the independent prognostic factors among SRCC patients. Receiver operating characteristic (ROC) curves were employed, and the area under the curve (AUC) was computed to assess and compare the discriminatory power and accuracy of various models that incorporated diverse LN status indicators. Based on the most favorable LN status indicators and other independent prognostic factors, a novel nomogram was devised to predict OS at 1, 3, and 5 years for SRCC patients. Decision curve analysis (DCA) and calibration curves were utilized in training and validation sets in an attempt to evaluate the potential utility and feasibility of the nomogram in predicting OS at 1, 3, and 5 years.

## Results

### Patient characteristics

Based on the established inclusion and exclusion criteria, 1663 patients diagnosed with colorectal SRCC were enrolled and randomly allocated into a training cohort and an interior validation cohort in a 7:3 ratio. Table 1 summarizes the baseline demographics of both cohorts. For subsequent analysis, the X-tile software was performed to calculate the optimal cutoff values for continuous variables such as tumor size, PLN, LNR, and LODDS. The derived thresholds were 39 mm, 5, 0.5, and 0.1, respectively. In the overall cohort, it was observed that the majority of SRCC patients were elderly males, accounting for over 50% of the population. The majority of SRCC cases occurred in the colon (86.2%) and were graded as grades III–IV (92.8%). Additionally, SRCC patients tended to present with advanced stage, primarily T3 or T4 (91.4%) and N2 (51.5%), and exhibited larger tumor sizes (76.2%). In terms of treatment, 53.9% of patients received chemotherapy, while 12.4% underwent radiotherapy.

**Table 1:** Demographic and clinicopathological characteristics of training and validation cohorts

	Training (*N* = 1164), *n*%	Validation (*N* = 499), *n*%	Overall (*N* = 1663), *n*%	*p*
Age				0.746
< 60 years	390 (33.5%)	172 (34.5%)	562 (33.8%)	
≥ 60 years	774 (66.5%)	327 (65.5%)	1101 (66.2%)	
Sex				0.060
Female	544 (46.7%)	259 (51.9%)	803 (48.3%)	
Male	620 (53.3%)	240 (48.1%)	860 (51.7%)	
Race				0.565
AI/API	102 (8.8%)	39 (7.8%)	141 (8.5%)	
Black	95 (8.2%)	35 (7.0%)	130 (7.8%)	
White	967 (83.1%)	425 (85.2%)	1392 (83.7%)	
Marital status				0.908
Married	984 (84.5%)	420 (84.2%)	943 (56.7%)	
Single	180 (15.5%)	79 (15.8%)	259 (15.6%)	
Primary tumor site				0.244
Colon	995 (85.5%)	438 (87.8%)	1433 (86.2%)	
Rectum	169 (14.5%)	61 (12.2%)	230 (13.8%)	
Grade				0.647
I–II	86 (7.4%)	33 (6.6%)	119 (7.2%)	
III–IV	1078 (92.6%)	466 (93.4%)	1544 (92.8%)	
Tumor size				0.932
< 3.9 cm	276 (23.7%)	120 (24.0%)	396 (23.8%)	
≥ 3.9 cm	888 (76.3%)	379 (76.0%)	1267 (76.2%)	
T stage				0.075
T1	40 (3.4%)	11 (2.2%)	51 (3.1%)	
T2	68 (5.8%)	23 (4.6%)	91 (5.5%)	
T3	661 (56.8%)	266 (53.3%)	927 (55.7%)	
T4	395 (33.9%)	199 (39.9%)	594 (35.7%)	
N stage				0.130
N0	311 (26.7%)	110 (22.0%)	421 (25.3%)	
N1	266 (22.9%)	119 (23.8%)	385 (23.2%)	
N2	587 (50.4%)	270 (54.1%)	857 (51.5%)	
M stage				0.070
M0	921 (79.1%)	374 (74.9%)	1295 (77.9%)	
M1	243 (20.9%)	125 (25.1%)	368 (22.1%)	
Chemotherapy				0.301
No/unknown	547 (47.0%)	220 (44.1%)	767 (46.1%)	
Yes	617 (53.0%)	279 (55.9%)	896 (53.9%)	
Radiation				0.794
No/unknown	1017 (87.4%)	439 (88.0%)	1456 (87.6%)	
Yes	147 (12.6%)	60 (12.0%)	207 (12.4%)	
PLN				0.371
< 5	622 (53.4%)	254 (50.9%)	876 (52.7%)	
≥ 5	542 (46.6%)	245 (49.1%)	787 (47.3%)	
LNR				0.624
< 0.5	765 (65.7%)	321 (64.3%)	1086 (65.3%)	
≥ 0.5	399 (34.3%)	178 (35.7%)	577 (34.7%)	
LODDS				0.654
< 0.1	810 (69.6%)	341 (68.3%)	1151 (69.2%)	
≥ 0.1	354 (30.4%)	158 (31.7%)	512 (30.8%)	

*AI* American Indian/Alaska Native, *API* Asian or Pacific Islander, *PLN* positive lymph node, *LNR* lymph node ratio, *LODDS* log odds of positive lymph nodes

### Identifying independent prognostic factors

**Fig. 2:**
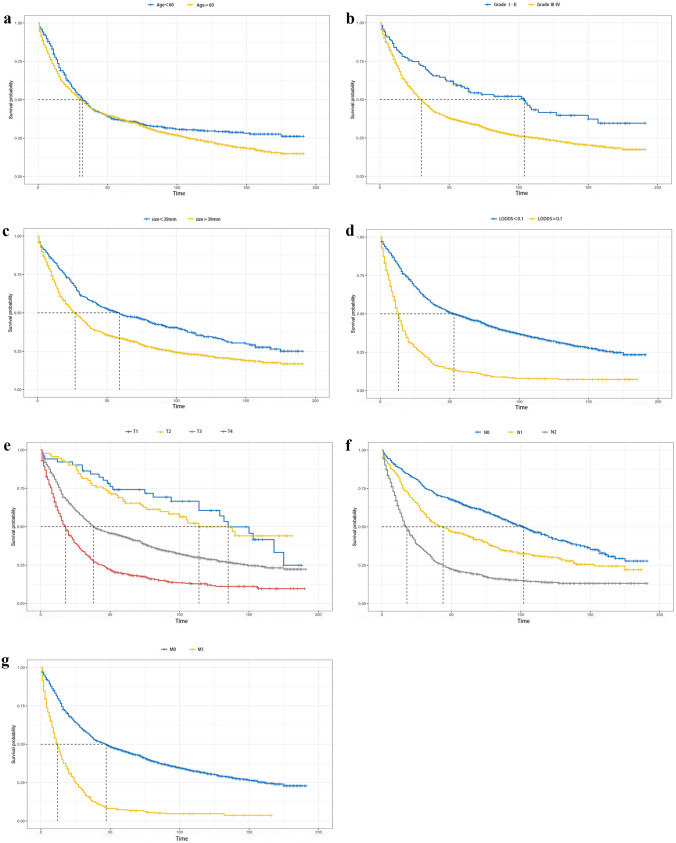
Kaplan–Meier estimates of overall survival for patients with colorectal SRCC after surgery according to **a** age, **b** grade, **c** tumor size, **d** LODDS, **e** T stage, **f** N stage, and **g** M stage. Abbreviations: SRCC signet ring cell carcinoma, LODDS log odds of positive lymph nodes

Detailed results of the univariate and multivariate Cox regression analyses in the training cohort are demonstrated in Table 2. Variables such as age, sex, racial background, marital status, primary tumor site, grade, tumor size, chemotherapy, radiotherapy, TNM stage, PLN, LNR, and LODDS were included in the univariate Cox analysis, among which age, grade, tumor size, TNM stage, PLN, LNR, and LODDS were found to have a statistically significant association with OS in colorectal SRCC patients. We further performed multivariate analyses and generated prognostic models incorporating various LN indicators respectively. Briefly, the independent risk factors for OS were narrowed down to N stage and LODDS in terms of LN status indicators, along with age, grade, tumor size, T stage, and M stage. Kaplan–Meier curves revealed significant statistical distinction in OS based on independent prognostic factors of SRCC patients (Fig. 2). To be specific, patients who were older and had advanced grades and TNM stages, larger tumor sizes, and higher LODDS values exhibited lower survival probabilities.

**Table 2:** Univariate and multivariate Cox regression analyses for OS in the training cohort

Characteristics	Univariable			Multivariable		
	HR	95%CI	*p*	HR	95%CI	*p*
Age						
< 60 years	Reference			Reference		
≥ 60 years	1.22	1.08–1.38	0.006	1.76	1.55–2.00	< 0.001
Sex						
Female	Reference					
Male	1.08	0.97–1.21	0.251			
Race						
AI/API	Reference					
Black	1.24	0.95–1.62	0.177			
White	0.89	0.73–1.08	0.316			
Marital status						
Married	Reference					
Single	1.13	0.97–1.32	0.204			
Primary tumor site						
Colon	Reference					
Rectum	1.11	0.94–1.30	0.292			
Grade						
I–II	Reference			Reference		
III–IV	1.90	1.48–2.44	< 0.001	1.59	1.24–2.05	0.002
Tumor size						
< 3.9 cm	Reference			Reference		
≥ 3.9 cm	1.58	1.37–1.81	< 0.001	1.23	1.06–1.42	0.021
T stage						
T1	Reference			Reference		
T2	0.91	0.56–1.46	0.733	0.87	0.54–1.39	0.619
T3	2.11	1.45–3.07	0.001	1.34	0.90–1.97	0.223
T4	3.53	2.41–5.16	< 0.001	1.64	1.17–1.66	0.042
N stage						
N0	Reference			Reference		
N1	1.57	1.32–1.86	< 0.001	1.39	1.17–1.66	0.002
N2	2.83	2.44–3.28	< 0.001	1.41	1.07–1.97	0.040
M stage						
M0	Reference			Reference		
M1	3.00	2.63–3.42	< 0.001	2.25	1.95–2.58	< 0.001
Chemotherapy						
No/unknown	Reference					
Yes	1.04	0.93–1.16	0.604			
Radiation						
No/unknown	Reference					
Yes	1.02	0.86–1.20	0.860			
PLN						
< 5	Reference			Reference		
≥ 5	2.45	2.18–2.75	< 0.001	1.30	1.00–1.69	0.099
LNR						
< 0.5	Reference			Reference		
≥ 0.5	2.51	2.24–2.82	< 0.001	1.15	0.85–1.55	0.459
LODDS						
< 0.1	Reference			Reference		
≥ 0.1	2.62	2.32–2.95	< 0.001	1.46	1.09–1.97	0.033

*AI* American Indian/Alaska Native, *API* Asian or Pacific Islander, *PLN* positive lymph node, *LNR* lymph node ratio, *LODDS* log odds of positive lymph nodes

### Comparison of different LN status indicators

**Fig. 3:**
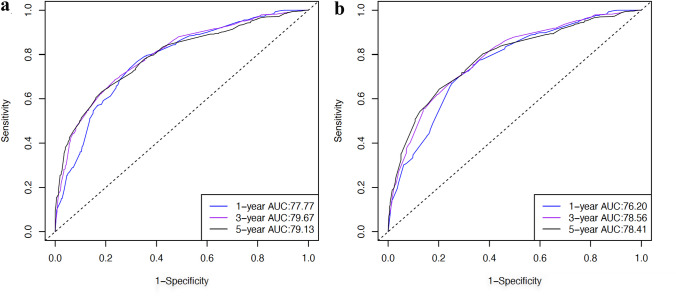
ROC curves for evaluating the discriminability between two models. Notes: **a** model incorporating both N stage and LODDS, along with other independent prognostic factors. **b** model incorporating N stage and other independent prognostic factors. Abbreviations: OS overall survival, ROC receiver operating characteristic, LODDS log odds of positive lymph nodes

The comparison of LN status indicators in the training cohort is shown in Fig. 3. Upon conducting multivariate Cox analyses, two models were evaluated for their predictive accuracy through ROC curves: one incorporating N stage alone and another incorporating both N stage and LODDS. Two models also included other independent prognostic factors. The AUC values in the latter model ranked higher (1-year AUC: 77.77; 3-year AUC: 79.67; 5-year AUC: 79.13) than the former one (1-year AUC: 76.20; 3-year AUC: 78.56; 5-year AUC: 78.41). Taken together, the results indicated that the selected model containing age, grade, tumor size, T stage, M stage, N stage, and LODDS offered superior predictivity for OS.

### Construction and validation of the nomogram

**Fig. 4:**
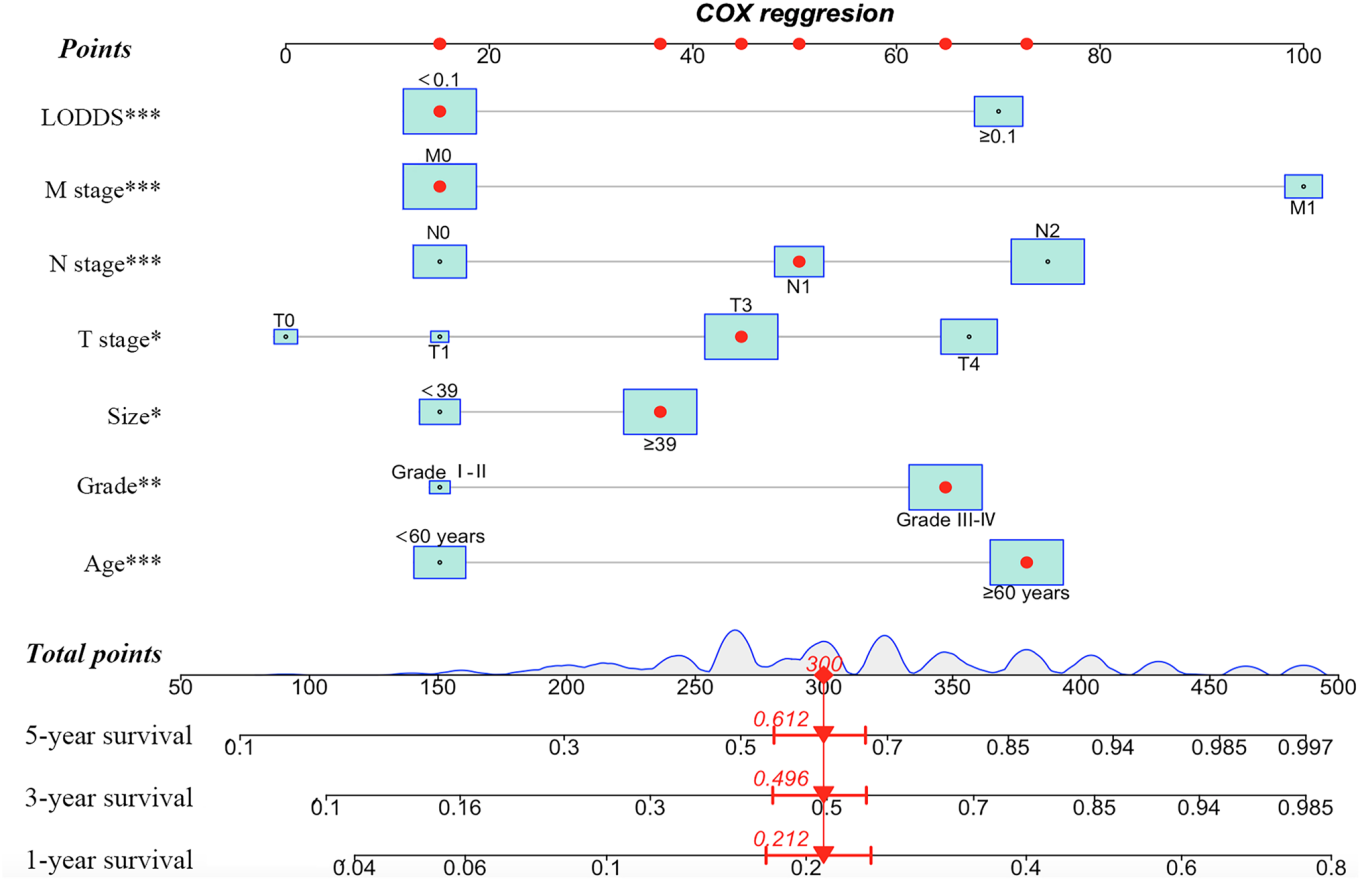
Nomogram for predicting 1-, 3-, and 5-year OS of patients with SRCC. Abbreviations: OS overall survival, SRCC signet ring cell carcinoma

**Fig. 5:**
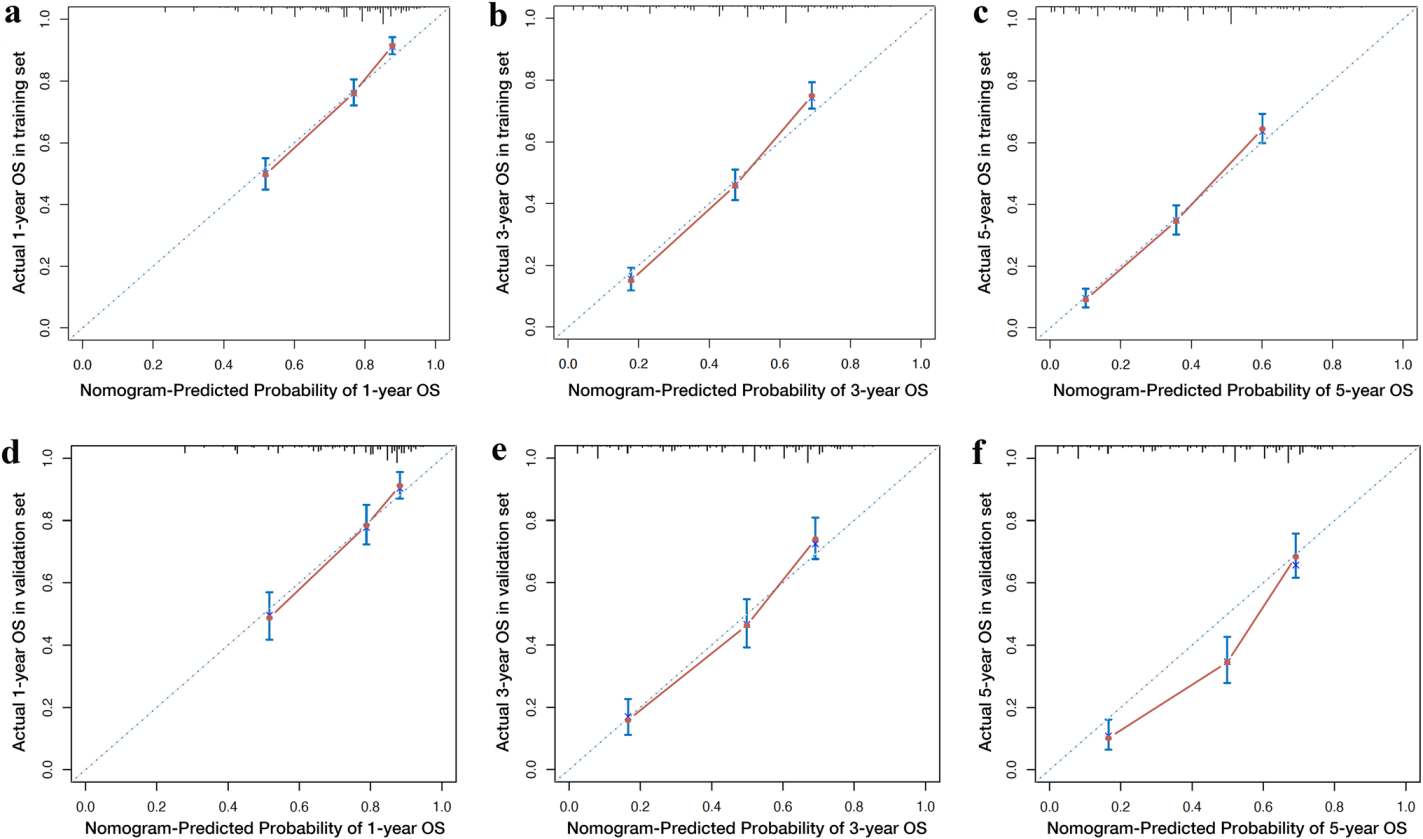
Calibration curves for evaluating the accuracy and reliability of the nomogram. Notes: **a**–**c** Calibration plots for predicting the 1-, 3-, and 5-year OS in the training set. **d**–**f** Calibration plots for predicting the 1-, 3-, and 5-year OS in the validation set. Abbreviations: OS overall survival

**Fig. 6:**
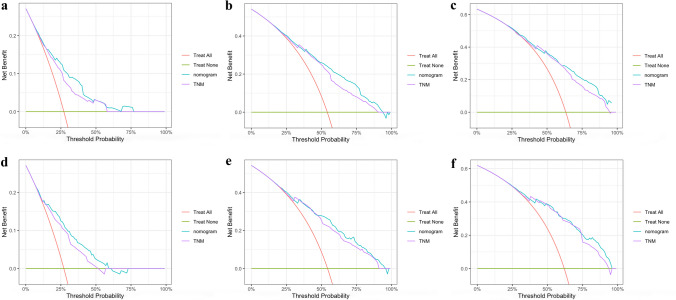
DCA curves for evaluating the clinical utility of the nomogram. Notes: **a**–**c** DCA curves of the nomogram and AJCC TNM staging system for predicting the 1-, 3-, and 5-year OS in the training set. **d**–**f** DCA curves of the nomogram and AJCC TNM staging system for predicting the 1-, 3-, and 5-year OS in the validation set. Abbreviations: DCA decision curve analysis, OS overall survival

We developed a nomogram based on the model containing both N stage and LODDS in the training cohort (Fig. 4). As a result, age, grade, tumor size, T stage, M stage, N stage, and LODDS were incorporated into the final nomogram for predicting OS. Additionally, calibration curves were generated to assess the concordance between predicted and actual probabilities of 1-year, 3-year, and 5-year OS in both training and validation cohorts (Fig. 5). These curves exhibited satisfactory agreement, highlighting the nomogram’s reliability. The DCA curves in both sets revealed that in comparison to traditional TNM staging, our nomogram offered superior net clinical benefits, exhibited excellent clinical utility, and effectively predicted the 1-, 3-, and 5-year OS of patients with SRCC (Fig. 6).

## Discussion

SRCC is a rare type of CRC, which exhibited a distinct behavior and reduced survival rates, despite tumor stage correction [9]. Previous studies have shown that SRCC had a propensity to localize in the proximal colon, to present in younger patients, to exhibit a worse grade of differentiation and increased likelihood of LNM, and to be found in advanced TNM stages [9, 10]. However, owing to the rarity of colorectal SRCC, most existing studies either have limited sample sizes or are confined to early-onset cases [11–13]. Mizushima et al. included 19 patients with primary colorectal SRCC identified from a database of 5884 surgically treated CRC patients at Osaka University Hospital and affiliated hospitals, of which lymph node involvement was observed in 14 patients, with overall 5-year survival rate in primary SRCC significantly lower at 24.1%, compared to 77.5% for well or moderately differentiated adenocarcinoma and 57.7% for poorly differentiated adenocarcinoma or mucinous carcinoma [14]. Another study by Nitsche et al. analyzed 160 colorectal SRCC patients out of a total of 28,056 patients in the catchment area of the Munich Cancer Registry, suggesting a higher frequency of poorly differentiated tumors, lymphatic invasion, and angioinvasion among SRCC patients [9].

In addition, several studies have already utilized the SEER database in an attempt to figure out the prognostic factors of colorectal SRCC and construct a nomogram for survival rate prediction [5, 15]. It is worth noting that traditional N staging proposed by AJCC TNM classification widely applied to the assessment of LN status is influenced by the number of total examined lymph nodes [16]. In other words, the prognosis of patients with 1 positive lymph node out of 1 harvested lymph node is of vital difference from patients with 20 positive lymph nodes out of 20 harvested lymph nodes, which casts doubt on the predictive value of LNR, a metric extensively studied in non-colorectal malignancies, including breast cancer, esophageal cancer, non-small cell lung cancer, and oropharyngeal cancer [17–20], but failing to further stratify patients with 0 or 1 PLN [21]. Meanwhile, LODDS, a novel LNM-related indicator for predicting cancer prognosis, has been put forward in previous studies where suggesting LODDS outperforms other LN status indicators in predicting the prognosis of bladder cancer, rectal cancer, small cell lung cancer, and so forth [22–24]. Since the presence of LNM is related to poor prognosis and determines the need for adjuvant therapy [25, 26], it is imperative to identify independent LN prognostic factors for SRCC and incorporate the indicators with the best predictive performances in an effort to construct a nomogram for SRCC patients.

In our study, a total of 1663 SRCC patients were retrieved from the SEER database, the majority of which were in grades III–IV, in advanced T and N stage, echoing previous findings [5, 27]. Kakar et al. postulated that SRCC patients tend to be younger and present with more advanced disease stages in comparison to mucinous carcinoma and conventional adenocarcinomas [28]. The aggressive biology of SRCC might be attributed to special molecular mechanisms, including a higher frequency of BRAF mutation, microsatellite instability-high (MSI-H), and CpG island methylator phenotype (CIMP) positive status [3, 29]. Regarding tumor size, our findings demonstrated that SRCC patients with tumors measuring less than 39 mm exhibited a lower prognostic risk. Alese, Zhou, and their team compared patients with tumor sizes ranging from less than 2 cm to greater than 10 cm, revealing that larger tumor sizes were associated with poorer survival outcomes, independent of other variables. This underscores the importance of determining adjuvant chemotherapy based on tumor size, particularly for patients who may be at high risk for recurrence or metastatic spread despite the absence of traditional high-risk features [30]. We noticed that patients above 60 years old were prone to worse prognosis in our research, contradicting Mauri’s results where early-onset CRC patients are characterized by a more advanced stage at diagnosis, compromised cellular differentiation, and higher frequency of SRCC histology [31]. This discrepancy calls for further research.

Through univariate and multivariate Cox regression analyses, age, grade, tumor size, T stage, M stage, N stage, and LODDS were identified as independent prognostic factors, free of PLN and LNR (*p* > 0.05), two indicators commonly studied to construct a nomogram and compare the predictive performances in colorectal, bladder, lung, and other cancers. ROC curves suggested that the model incorporating both N stage and LODDS, together with other independent prognostic factors, had higher AUC values. In the final model, age, grade, tumor size, T stage, M stage, N stage, and LODDS were utilized to assess the OS in SRCC patients. Then, internal validation cohorts further validated the nomogram, with calibration curves demonstrating stable linearity and effectiveness and DCA curves demonstrating consistent and substantial net benefits, supporting the clinical utility of the nomogram in predicting OS for SRCC patients. Taken together, our nomogram outperformed the typical TNM staging system in predictive accuracy and clinical validity. It was speculated that the TNM staging is only suitable for preoperative evaluation, whereas our nomogram offers greater precision in predicting standing survival outcomes following surgical intervention. Our nomogram serves as a valuable tool for survival consultation, a perpetual concern shared by medical professionals and patients alike. It also offers guidance in clinical decision-making and treatment allocation. Given that patients with higher aggregate scores are anticipated to face a less favorable prognosis, it is advisable for these individuals to undergo supplementary treatment and rigorous follow-ups in the future.

To the best of our knowledge, the current study is the first to meticulously incorporate four LN status indicators, compare the prognostic performances across models, and preliminarily explore the merger of both LODDS and N stage to enhance the existing AJCC TNM classification, finally constructing a nomogram with satisfactory clinical utility which visualizes the 1-year, 3-year, and 5-year OS evaluations for colorectal SRCC patients. Compared with existing prognostic nomograms for SRCC patients, our nomogram is applied to patients in all TNM stages. It enriched the dimensions of examining the state of LNM and circumvented the issue of the inaccuracy and inappropriateness of using solely N stage to represent the LN status. Through ROC curves, we quantitatively compared AUC values between models, and finally validated the predictive superiority of our nomogram. Nevertheless, several limitations must be acknowledged. Firstly, the SEER program collects data from 18 states throughout the United States, resulting in some extent of generalizability. External validation with sufficient sample size is urgently needed to ensure the applicability of our nomogram in the future. Secondly, the SEER database failed to provide possibly crucial data such as tumor marker CEA, microsatellite stability, BRAF, specific chemotherapy drugs, and specific radiotherapy dosage. Thirdly, we opted to use the 6th edition of the AJCC TNM staging system to guarantee the sample size instead of using the newer edition or re-grouping the TNM information based on the 8th AJCC TNM staging system which may decrease accuracy to some extent. Finally, the relationship between total points and specific following treatment such as adjuvant treatment cannot be easily determined as it needs in-depth and meticulous research. In future research, our nomogram should be optimized to have more applications such as studying the relationships between total points and tumor microenvironment, immunity, and so forth by introducing other databases or enriching the data source, which may directly or indirectly benefit the clinical practice in significant ways.

## Conclusion

In conclusion, SRCC is a rare type of CRC with a relatively worse prognosis. This study confirmed that incorporating both LODDS and N stage as LN status indicators has better predictive accuracy compared with only taking traditional N stage into account for colorectal SRCC patients after surgery. A novel nomogram containing age, grade, tumor size, T stage, M stage, N stage, and LODDS for predicting OS was established based on the SEER database and successfully validated in the interior validation cohort, promising more accurate therapeutic decisions and personalized follow-up management for colorectal SRCC patients.

## Data Availability

No datasets were generated or analysed during the current study.
